# Two Types of Formalism of the Rule of Law

**DOI:** 10.1093/ojls/gqab039

**Published:** 2021-11-03

**Authors:** Konatsu Nishigai

**Keywords:** adjudication, rule of law, common law, Dicey, jurisprudence

## Abstract

The aims of this article are twofold: (i) to propose an explanatory framework, focusing on law-making acts, for accounting for whether the formal requirements of the rule of law are fulfilled; and (ii) to propose two further models within this framework. One model, which I call ‘rulebook formalism’, pertains to Parliament’s law-making acts; another model, which I call ‘rights formalism’, concerns the courts’ law-making acts. This distinction results from the different modality of law, ie the different natures of law-making acts. Drawing on speech act theory, I give a general account of the formal requirements as the success conditions of law-making acts. Then, applying this framework, I discuss the formal requirements for Parliament’s law-making acts and the courts’ law-making acts respectively.

## 1. Introduction

Certain requirements must hold for the rule of law to function. Lon Fuller enumerates such requirements, including the law being general, open, prospective, clear and without contradiction, not requiring the impossible and being relatively stable.^1^ Joseph Raz, in his 1977 article, argues that this list represents the formal concept of the rule of law, as distinct from the substantive concept of the rule of law.^2^

As Fuller presented the list as the requirements for Rex to make law, this list is legislation-centred. When laws are made by legislation, they are typically explicit and general rules that mandate or prohibit certain actions of the norm subjects. Because Fuller’s list of requirements primarily focuses on the laws made by legislation, the list does not account for other types of law-making acts, namely laws arising from officials’ law-applying practices, including judicial law making. Being aware of this problem, in his 2019 article, Raz admits that the existing requirement list is insufficient and greatly expands the list by adding new items.^3^ In effect, the new items added by Raz seem to constitute a new list as distinct from the list endorsed by Fuller. I will return to Raz’s new list in section 3.

The purpose of this article is to propose two variations of the list of formal requirements of the rule of law, one of which accounts for statutory law, while the other accounts for the common law. The latter is presented as the application of the underlying considerations behind Fuller’s list to judicial law-making, which was outside the scope of consideration in his discussion. I argue that the different success conditions of Parliament’s and the courts’ law-making acts necessitate different lists of formal requirements of the rule of law. I also argue that this list is the formal requirement of the rule of judge-made law (rights formalism), as opposed to the formal requirements of the rule of legislated law (rulebook formalism).

To this end, I first discuss the implication of acknowledging different types of law-making acts as to whether the law is a modal or functional kind. Then I consider why the analysis of law’s form must focus on law-making acts and why such acts vary. After explaining how Fuller’s list can be understood in terms of the success conditions of commands in speech act theory, I enumerate the success conditions for judicial law-making acts by comparing judicial law-making and legislation. In section 2A, I discuss the nature of the formal requirement in terms of law’s modality. In section 2B, I introduce a new explanatory framework focusing on law-making acts, by showing that the current widely accepted list of requirements of the rule of law is best understood as a list of success conditions for Parliament’s law-making acts and law’s features postulated by their success. Section 3A discusses the expansion of the current list of requirements in light of judicial law-making acts. In section 3B I propose a model I call ‘rights formalism’, which incorporates judicial law-making acts into law’s modality. In section 2C the specific additions to the list of items that rights formalism demands are enumerated, by examining the success conditions for the courts’ law-making acts.

## 2. The Formal Requirements of the Rule of Law

What is the form of the rule of law, or law’s form? In this section, by examining the law’s modality, I argue that the formal requirements are the success conditions for law-making acts. Exploring the current debates as to whether the law is a modal or functional kind, I first explain why acknowledging a new type of law-making act and proposing a new list of the formal requirements does not contradict the formalism, endorsed by John Gardner, which sees the form of law as law’s modality. Drawing on speech act theory, I then show that Fuller’s list is best understood as a set of requirements for law’s modality postulated by Parliament’s successful law-making acts.

### A. Law’s Form: The Concept and the Items in the List

According to Fuller, laws should be general, open, prospective, clear, without contradiction and relatively stable, and should not require the impossible.^4^ Lon Fuller presents this list as the law’s desiderata. In his 1977 article, Raz highlights the basic idea underlying these requirements, which is the law’s capability of ‘guiding the behaviour of its subjects’.^5^ He says that this conception, the capacity for action guiding, is ‘a formal one’ because ‘It says nothing about how the law is to be made: by tyrants, democratic majorities, or any other way. It says nothing about fundamental rights, about equality, or justice’.^6^ Raz’s account proved very influential; although Fuller himself presented the above list as the ‘inner morality’ of law, in current literature, his list is now associated with a ‘formal’ or ‘legalistic concept’ of the rule of law,^7^ as distinct from the substantive concept of the rule of law. Raz also explains that the value of the rule of law is similar to the value of producing a sharp knife. He notes that ‘A knife is not a knife unless it has some ability to cut’.^8^ Being a sharp knife does not mean that it will always be used for a good purpose. The same applies to the rule of law. The fact that law has the capacity to guide action does not necessarily mean that law with this capacity will be used only for good purposes. Action guiding is merely a minimal function that is *necessary* for the law to serve as a tool in the first place.

While Gardner criticises the distinction in the literature between form and substance as misleading,^9^ he argues in favour of understanding the formal concept of the rule of law in terms of law’s modality, invoking Leslie Green’s characterisation of law as a modal kind, as opposed to a functional kind.^10^ Underlying the distinction between modal and functional kinds is a debate on the nature of artefacts.^11^ A functional kind is a type of entity whose presence is determined by its function. A modal kind refers to a type of entity that is identified by its necessary features. Artefacts are a subclass of modal kind, in the sense that their necessary features are postulated by the human acts of creating them. For instance, a printer driver is a modal kind. As Green explains, if a driver cannot run a printer because of a bug, we do not say that it ceases to be a printer driver. This is because we know that ‘it was designed for its function and, if fixed, still may perform it’.^12^ The function of a printer driver is its *capacity* that is postulated by the human actions of creating it.

The upshot of acknowledging law as a modal kind is to deny teleological arguments: we cannot say ‘It is not the law because it is not liberal’ or ‘It is not the law because it is not committed to protecting human rights’. Law is a social artefact whose capacity, action guiding, is postulated by the human actions of creating it. Laws, like screwdrivers and printer drivers, remain laws even when they fail to perform their intended functions, since they were created for the purpose of being laws. Failing to achieve liberal ideals does not exclude such a law from membership of the category of law. Law’s modality is merely ‘minimal social functions’ and a ‘technique’ to achieve various types of political ideal.^13^

However, the fact that law’s modality is minimal functions does not mean that the items outside Fuller’s list are irrelevant to its modality. For there are more than two ways for a system to have a functional capacity to guide actions. I argue that the distinction between a modal kind and a functional kind does not work, since the two are not mutually exclusive. Law is a modal *and* functional kind.^14^ Many artefacts are functional kinds, and there are multiple ways for them to have their supposed functional capacity. Take a printer driver as an example. A printer driver is a program that can be written in numerous programming languages, and the ways of composing a printer driver using any language also vary. If a programmer intended to create a printer driver but forgot to write the bulk of the essential lines of the program, then we would say ‘She failed to create a printer driver’, which means that she did not write a printer driver despite her intention to do so. But the lines essential for writing a printer driver differ greatly depending on which programming language is used. To specify requirements for a printer driver, we first need to determine which language the driver will be in.

In the case of law, its supposed capacity of guiding actions is conferred by the success of law-making actions. Fuller discusses law’s malfunctions in terms of the unsuccessful attempts to create law. Unlike certain modal kinds, such as water, law’s necessary features are not postulated by its chemical molecular structure, but by its functional capacity of guiding actions. Fuller’s list is not the only list of requirements of the rule of law. Acknowledging this is compatible with acknowledging the law as a modal kind and with acknowledging the ‘form’ of the rule of law. If there are different types of law-making actions, they require different sets of success conditions.

As for the general explanatory framework of the rule of law, we can accept that the law is a modal kind in the sense that the rule of law pertains to necessary features and conditions that are postulated by law-making acts. I use the terms ‘formalism’ and ‘law’s form’ to denote the concept of the rule of law which is based on the view that sees law as a modal kind in this sense. Law’s modality is postulated by the actions of creating it. But law is a tool-type artefact, whose necessary capacity is endowed by law-making acts. There are several ways of making general rules understood. Acknowledging the law being a modal kind does not rule out the possibility that each member of the category belongs to a different subcategory of law’s modality, for there can be more than two ways for law to have its supposed functional capacity, the guidance of action. Regarding *which items* should be included in the requirement list, we must acknowledge that there are different possible ways of making law, and that law’s necessary features and the success conditions for making law depend on which type of speech act a law maker uses in each institutional setting. We can start by looking into the current requirement list to see what type of law-making acts postulate the conditions and features as the items in the current list.

### B. The Success Conditions for Law-Making Acts

Law-making acts are speech acts, ie actions performed by uttering words.^15^ By uttering the words of statutes, albeit figuratively, Parliament commands, prohibits and permits (I will henceforth simply call the utterances ‘commands’ in terms of speech acts).^16^ Commands, in John R Searle’s terminology, are a type of *directive*, whose characteristic aim is to guide actions, ie to try to get people to do or not do something.^17^ There are different ways to guide others’ actions via directives. For example, requesting, begging, pleading and advising are also members of the class of directives and therefore have the same characteristic aim as commanding. However, they may have their different modes of achieving this aim.^18^ For instance, the command achieves the characteristic aim, ie guiding action, ‘by way of invoking the position of authority of the speaker’.^19^ Crucially, a command can fail to guide actions in the way its speaker intends. In the literature on speech acts, these mishaps are discussed in terms of the success conditions for a speech act.^20^

The formal requirements for the rule of law endorsed in the current literature are best understood as the success conditions for *commands*, namely: (i) the speaker’s authority; (ii) the hearers’ ability to do what they are told; and (iii) the hearers’ comprehension.

The speaker’s authority requires promulgation^21^ as a success condition for Parliament’s legislative acts. The command has its own mode of achieving the guiding of action, one which requires the speaker’s authority.^22^ In a modern state, the specific procedure, rather than the identity of a particular person, indicates the authority of the agent. In this case, the breach of this type of important protocol strips the speaker of the relevant authority, which leads to the unsuccessful performance of an intended command. Promulgation is one such protocol.

The hearers’ ability to do what they are told requires the command to be prospective and consistent. When commanding, the speaker must presuppose this ability of the hearers. Suppose an authority commands people not to have sunbathed last Sunday. In this case, the commander cannot be said to have presupposed the hearers’ ability since, clearly, people cannot change their past behaviour. Thus, this command fails. Being prospective is a *propositional content condition* of a command.^23^

The hearers’ comprehension requires clarity and promulgation. Although what a law commands does not need to be crystal clear in every case, it should be ‘sufficiently clear to guide an individual’s conduct’.^24^ If what Parliament says is hopelessly unclear, the supposed legislative act cannot successfully guide actions. This is Fuller’s fourth desideratum on the clarity of law.^25^ Furthermore, without announcing (promulgating) a law, people cannot know what Parliament wants them to do, thus the hearers cannot comprehend the command.

In sum, the list of formal requirements is the list of the success conditions of Parliament’s law-making acts. The success conditions for Parliament’s legislative acts as commands include:

- the speaker’s authority;
- the hearers’ comprehension (eg clarity, promulgation); and
- the hearers’ ability to do what they are commanded (eg prospectivity, consistency).

Fulfilling the action-guiding function is the characteristic aim of Parliament’s law-making acts. Laws produced by Parliament’s successful performance of a command are clear, prospective and consistent, and therefore have the virtue of guiding action. These virtuous features are necessary for the successful performance of the command *qua* law-making act.

I will call the law’s modality assumed in the current formal requirement list ‘rulebook formalism’.^26^ For rulebook formalism, Parliament’s law-making acts postulate the modality of law. However, there are different ways to make general rules understood. I consider a different type of law-making act, since the formal requirement list discussed so far is not sufficient; it only focuses on a single type of law-making acts—explicit commands. This list cannot account for the formal rule-of-law requirements for institutions that generate law by a different speech act. In section 3, I will discuss how the current list can be expanded to account for law-making acts by a law-applying institution.

## 3. The Courts’ Law-Making Acts and Their Formal Requirements

In section 2A, I suggested that there are different ways of making general rules understood. In section 2B, I discussed one way of achieving this, via an explicit type of law-making act—commands—and enumerated the requirements for their success. In this section, I consider a different way of making law. I propose that rights formalism explains the modality of law postulated by the successful performances of the courts’ law-making acts. In subsection A, I discuss the expansion of the list of formal requirements by accounting for necessary conditions and features that are postulated by well-ordered law-applying practices. To this end, subsection B articulates the nature of the courts’ law-making acts. To spell out the items that should be added to the list of formal requirements, in subsection C I discuss the success conditions for the courts’ law-making acts. I argue that the courts’ law-making acts are *indirect commissives*—a type of promise performed implicitly. The different nature of the courts’ law-making acts compared to Parliament’s requires different conditions for their successful performance, namely: the allocation of authority to ordinary tribunals; the preservation of the full scope of jurisdiction; and the force of their precedents.

### A. Parliament’s Law-Making Acts and the Courts’ Law-Making Acts

Albert Venn Dicey’s understanding of the rule of law exemplifies the law’s modality postulated by the actions of the courts. Dicey names three requirements: the absence of arbitrary power; equality; and judicial decisions in particular cases resulting in judge-made law. By the absence of arbitrary power, he means that ‘no man is punishable or can be lawfully made to suffer in body or goods except for a distinct breach of law established in the ordinary legal manner before the ordinary Courts of the land’.^27^ This situation contrasts with the exercise ‘by persons in authority of wide, arbitrary, or discretionary powers of constraint’.^28^ The rule of law also requires, according to Dicey, that ‘every man, whatever be his rank or condition, is subject to the ordinary law of the realm and amenable to the jurisdiction of the ordinary tribunals’.^29^ He refers to this idea as ‘legal equality’, ie ‘the universal subjection of all classes, to one law administered by the ordinary Courts’.^30^ As a result of this, all the officials ‘are as responsible for any act which the law does not authorise as is any private and unofficial person’.^31^ Dicey’s rule of law requires, as the third element, that ‘the general principles of the constitution … are … the result of judicial decisions determining the rights of private persons in particular cases brought before the Courts’.^32^

Paul Craig explains Dicey’s understanding of the rule of law as a formal one. According to him, the arbitrariness that Dicey mentions results from either a sheer lack of or severe defects in legal rules, namely, the lack of legal foundation and the vagueness or the lack of clarity of the legal rules in question.^33^ Craig also explains that Dicey’s second meaning, equality, requires ‘the formal access to the courts’.^34^ Regarding Dicey’s third meaning, he acknowledges that it ‘does not sit easily with the previous two’.^35^ He puts forward the view that Dicey, by putting emphasis on common law, is merely saying that ‘if you wished to protect such rights then the common law technique was better than that employed on the continent’.^36^ This interpretation avoids acknowledging that the protection of rights is a necessary capacity of law—it is merely a contingent feature.

However, Dicey’s emphasis on the common law technique would be far too strong if it were a contingent feature of law. In view of the rise of the welfare state at the beginning of the 20th century, Dicey finds ‘a marked tendency towards the use of *lawless* methods’ in a pattern of legislation which was designed to oust the courts’ jurisdiction.^37^ Lawlessness for Dicey results from eliding the role of the ordinary courts. As to equality, for Dicey, citizens’ mere access to the courts does not suffice to secure the rule of law, but ‘the rule of the High Court’^38^ does. For Dicey, the ordinary courts, not administrative tribunals, need to hold this full scope of jurisdiction, which JWF Allison calls ‘jurisdictional equality’, ie ‘the subjection of all to the same jurisdiction’.^39^ As to the protection of rights, what Dicey says is that protecting rights is the special institutional function, whose generalisation results in the common law constitution.^40^ That is, if the courts do not protect rights in each case, there is no constitutional law in the UK.

Furthermore, Dicey’s emphasis on ordinary law implies more than what is necessitated by Parliament’s successful law-making acts. By ‘ordinary law’, Dicey means that officials and private individuals are under the same general rules.^41^ Put differently, it is the generality of law in terms of its applicability to persons.^42^ The larger the class of persons to which a law applies, the more general the law is in this respect. Fuller, in his first desideratum, suggests that generality is inherent in command-type legislative acts as long as they guide action. According to Fuller’s first desideratum, ‘the law must act impersonally … its rules must apply to general classes and should contain no proper names’.^43^ Dicey’s ‘ordinary law’ is more demanding than Fuller’s generality. Dicey’s ‘ordinary law’ not only means anonymous application; it also means the same application to different groups of people. Let us suppose that there is a law solely applicable to ‘officials’, a class of anonymous persons. In this case, Fuller’s first desideratum is met since the addressees of this law are ‘officials’, not ‘Mr Jones’. But Dicey’s ‘ordinary law’ requirement is breached because it necessitates applying the same rule to officials and private individuals. The level of generality with which Dicey is concerned is more demanding than Fuller’s notion of generality as anonymity. The level of generality that Dicey assumes cannot be explained in terms of the success conditions of Parliament’s law-making acts.

Fuller attempted to deal with the issue of law-applying institutional practice in the desideratum ‘congruence between official action and declared rule’, which Gardner renames as the desideratum that law should be ‘upheld by officials’.^44^ Fuller admits that this desideratum is ‘the most complex of all the desiderata that make up the internal morality of the law’.^45^ The congruence, according to Fuller, might be destroyed by a wide range of activities, such as ‘mistaken interpretation, inaccessibility of the law, lack of insight into what is required to maintain the integrity of a legal system, bribery, prejudice, indifference, stupidity, and the drive toward personal power’.^46^ Nevertheless, statutory rules, however successfully created, cannot eliminate discretionary powers or close all loopholes in advance. However, preventing these calamities, put in Raz’s terms, depends on the question of how much discretion officials should have.^47^ Mere congruence between officials’ actions and rules successfully created by the legislative institution cannot eradicate corruption or avoid ineptitude.

Raz, in his 2019 article,^48^ acknowledges that ‘the rule of law doctrine has been inadequately identified. Perhaps simply only part of it was stated.’^49^ Based on this observation, he greatly expands the original list of the formal requirements by adding the following: the publicising of the reasons for decisions; the fair and unbiased process of decision making; the reasonableness of decisions; conventions raising the presumptions ‘that observing them serves the interest of the governed and that the officials who follow them act in the interests of the governed’; and public culture embodying the doctrine of the rule of law.^50^ The expansion of the list should, in principle, be welcomed, as the original list cannot account for the normative aspects of law-applying practice. However, we still need to consider the following points. Although very few people would assume that law-applying officials should act contrary to the new requirements that Raz proposes, he does not provide detailed justification for the inclusion of the new items. Furthermore, in order to expand the list, Raz introduces a new ideal of ‘the interests of the governed’ as another core feature of ‘act[ing] as a government’.^51^ According to our understanding of law’s modality, items on the list are supposed to be conditions or features that are postulated by acts that create laws. Does acting as a government relate to the creation of particular laws? Or is it an teleological argument? In what sense can Raz’s new requirements be added to the list, if the new items and the existing items are inspired by two different ideals: ‘the interests of the governed’ and the guidance of action?

Fuller’s strategy to spell out the requirements for legislative acts cannot account for the ‘upheld by officials’ desideratum. It is not simply because officials’ actions are not the legislator’s. What we think of as well-maintained law-application practice is more than mere congruence between statutory laws and the practice of upholding them, for it is often the case that a proper ‘application’ cannot be decided by the statutory law itself. However, acknowledging that there are laws made by nobody immediately contradicts the account of the formality of the rule of law as law’s modality, ie a set of necessary conditions and features postulated by human acts of creating it. The most feasible strategy is to acknowledge another agent creating law in terms of the application of law.

We now need to take the second step. The first step, as discussed in the last section, was to focus on law-making actions as the law’s modality. As we saw, the current list of formal requirements can be explained in terms of the success conditions for *commands*. The next step is to introduce implicit law-making acts into the formal framework. Acknowledging implicit law-making acts enables us to spell out the formal requirements as to law-applying practice, ie the modality of law that successful law-applying practice necessitates. I focus on the acts performed by the courts for two reasons. First, although the courts never explicitly admit it, they have significantly wide discretionary power.^52^ Second, the courts have the final say on other institutions’ law-applying practices. Also, as Hart highlights, this final say extends to law-making acts by Parliament: even if what the courts said was wrong by Parliament’s standards, the courts’ decisions are final.^53^ Below, I discuss the mode of the courts’ law-making acts, which constitute a modality of law. Then, in subsection C, I enumerate the items to be added to the list of formal requirements of the rule of law in light of the success conditions of the courts’ law-making acts.

### B. Law-Making Acts as Indirect Commissives

Two views dominate the debate over the nature of adjudication.^54^ One view is called the declarative theory of law, typically found in William Blackstone’s work. According to this view, judges are ‘not delegated to pronounce a new law, but to maintain and expound the old one’.^55^ It assumes that ‘They are the depositaries of a body of customary principles which have only to be applied to each new case as it arises’.^56^ John Austin opposes this view and ridicules it as claiming that ‘judiciary or common law is not made by [judges], but is a miraculous something made by nobody’.^57^ For Austin, this view is a ‘childish fiction’; he prefers to see veritable law-making power in judicial decisions, ‘a certain power of making rules for cases not provided for previously’.^58^

Dicey acknowledges that judge-made law ‘really ha[s] the force of law and [is] made by the Courts’ and calls ‘English Courts’ ‘the English Legislature’.^59^ Dicey and Austin, however, differ in their evaluations of such laws. For Austin, an enthusiast of codification, judge-made law is nothing but a tragedy because it is *ex post* law.^60^ But Dicey is not so cynical; he believes that professors can portray general rules of the common law and ‘reduce the mass of legal rules to an orderly series of principles’.^61^ In contrast to a country with vigorous codification, which communicates rules by explicitly bidding so, Dicey argues that a country wherein the law is predominantly the product of the courts has a different way of identifying law. He says,

in Belgium individual rights are *deductions* drawn from the principles of the constitution, whilst in England the so-called principles of the constitution are *inductions* or *generalisations* based upon *particular decisions pronounced by the Courts as to the rights of given individuals*.^62^

Dicey says that the common law is understood by ‘inductions’, that is, by making hypotheses from the decisions made by judges on rights of particular individuals under particular circumstances. The common law requires the hearers to use backward reasoning^63^ to find general rules, a key point of contrast with the continental law.

To see what Dicey means by ‘inductions’, it is useful to first consider a supposedly standard type of legal reasoning—deductions. According to Neil MacCormick, deductive or subsumptive reasoning in law is characterised by three steps:

1. ‘postulat[ing] a general hypothetical rule’;
2. ‘establish[ing] facts in a particular case subsumable within the rule’s hypothesis’; and
3. ‘draw[ing] the logical conclusion for the particular case from rule plus facts’.^64^

If a relevant general rule is given, this is how we (and judges) deduce (3) for each case, which is the same process in both common law and civil law systems. But in a common law system, we need to work out not only (3) but also, occasionally, (1) by backward reasoning of the judges’ reasoning as seen in past authoritative decisions. In a codified system, which strives to establish a sophisticated system of knowledge, general rules are supposed to be readily accessible since codes are systematised as general rules under a few legal maxims, wherein the concepts are defined as explicitly as possible. In a common law system, however, general rules are not always given as handy codes, meaning that some extra work is needed to obtain general rules (1). In their decisions, the courts explicitly describe facts (2) and give remedies (3). If their decisions in similar situations display certain dispositions, we can construe general rules (1) as the generalisation of such dispositions. The courts do *not* explicitly postulate general rules (1) by explicitly commanding or declaring them. However, thanks to the hearers’ backward reasoning, Dicey observes that ‘there exists a whole body of law called judge-made law’, although the courts are there to establish facts and give remedies.^65^ Dicey calls this the paradox of judge-made law.^66^

The questions we must ask here are the following: in what conditions can we infer general rules from the courts’ acts of describing facts and giving remedies? What kind of acts do the courts perform to make such general rules understood? In order to answer these questions, I first look into the nature of the courts’ decision giving, which I call ascription, following Hart’s earlier work. Next, I look into another act, which is performed implicitly by way of ascription and gives rise to our expectation of *stare decisis*. Following Searle, I will call such an act an *indirect commissive*. Lastly, I discuss the success conditions of the courts’ indirect commissives.

When the courts give decisions, their decisions have special normative force. Kelsen argues that judicial decisions create individual norms.^67^ Dicey calls judicial decision giving the enforcement of rights. According to Dicey, what characterises the British constitution is the ‘inseparable connection between the means of enforcing a right and the right to be enforced’.^68^ The British constitution focuses ‘intently on providing remedies for the enforcement of particular rights or … for averting definite wrongs’.^69^ Giving remedies, for Dicey, is not a mere use of force. It is the act of giving a normative decision, which Dicey calls the enforcement of rights. This act—or, more precisely, the accumulation of these acts—is the very source of the common law.

In his earlier work, Hart called this act of giving a normative decision *ascription*.^70^ The courts’ statements of facts are always written in a descriptive form as if they were merely evidencing the fulfilment of the conditions of a general legal concept. However, in reality, the courts are performing an ascription of rights and responsibilities by way of describing the fact. This ascriptive function is typically found in lawyers’ language. This language, including the language used in judgments given by the courts, uses modals suitable for descriptive purposes, but in fact the courts’ statements are ascriptive. Hart argues that statements of certain facts in the courts’ decisions are examples of such language with an ascriptive function. For example, the statement ‘This is yours’, uttered by one of two brothers to the other after fighting over a toy, is not a mere description. That is, it does not describe a pre-existing state of ownership; the speaker, by making the utterance, intervenes in the world and ascribes the property right in the toy to his brother, thereby conducting a transaction.

Hart’s ascription is, in Searle’s terminology, a type of speech act called a *declarative*, whose ‘successful performance … guarantees that the propositional content corresponds to the world’.^71^ Naming and christening are also examples of declaratives. The characteristic aim of declaratives is ‘to change the world by saying so’.^72^ Hart states that ascription is the ‘primary function’ of the language used by the courts, although the courts perform the act of ascription by uttering descriptive statements. In Searle’s terminology, speech acts that describe facts are *assertives*, whose characteristic purpose is ‘to commit the speaker … to the truth of the expressed proposition’.^73^ That is, the courts are performing declarative acts by performing assertive acts.^74^ What appears to be a merely descriptive practice is in fact a prescriptive normative intervention. I will use Hart’s term ‘ascription’ to refer to this normative intervention.

The courts’ act of ascription entails specific normative statuses held by the people concerned in each case. Hart calls these statuses ‘rights and responsibilities’, while Dicey refers to them as rights and duties.^75^ In the current discourse, it is more common to put these normative statuses in Hohfeldian terms, namely, to speak of claim rights, privileges (or liberties), duties and no-rights. By describing facts and giving remedies, the courts in fact ascribe these statuses to people involved in each case. Here, I use a more general term ‘deontic statuses’ to refer to this class of statuses the courts ascribe to people.

There are also presumptive deontic statuses prior to judicial decisions. In most cases, these deontic statuses are recognisable without actual judicial ascription. If the courts show a disposition to ascribe certain deontic statuses in certain situations, the hearers can anticipate this beforehand, even without going to court. I will henceforth call these statuses *presumptive deontic statuses*, in order to distinguish them from the deontic statuses which are judicially ascribed in actual decisions. Dicey’s definition of rights can be understood primarily as referring to such presumptive deontic statuses. He defines a ‘legal right’ as ‘a man’s capacity for influencing the conduct of others … by means of the opinion or force of the State’.^76^ It is a status that is construed as a *capacity* of an agent prior to a judicial ascription. Under a certain set of conditions, the courts’ normative interventions as ascriptions raise the hearers’ expectation that the courts ascribe certain deontic statuses to individuals in certain situations. From the hearers’ point of view, this is an expectation as to their own presumptive deontic statuses. People understand presumptive deontic statuses as prescriptive and binding even without the actual involvement of the judicial process, as explained later. Judicial ascriptions are particular and often past-oriented. However, through the expectation of *stare decisis*, these historical decisions give rise to presumptive deontic statuses even for people not yet involved in the judicial process.

The courts raise our expectation of standardised ascriptions in future similar cases, by ascribing deontic statuses to particular persons in each case. In terms of presumptive deontic statuses, the courts’ ascription of deontic statuses to particular persons functions as their commitment to the same course of action in similar cases. In Searle’s terminology, this means that the courts perform indirect commissives^77^ by raising the hearers’ expectation of the courts’ future standardised ascriptions. Such commissives are indirect^78^ insofar as they are performed in addition to and by means of another type of speech act. ‘I can do that for you’,^79^ from one of Searle’s abundant examples, shows how a speaker can perform an indirect offer or a promise by uttering a sentence about the speaker’s ability. Likewise, a speaker can indirectly promise by uttering a sentence about her sincerity, eg ‘I intend to do it for you’.^80^ Even when the courts do not explicitly state their ability, the fact that they perform ascriptions indicates their ability to ascribe similar deontic statuses in future similar cases. Furthermore, the backdrop against which judicial reasoning (the courts’ interpretation of statutes or their identification of common law principles) becomes prescriptive is their sincerity in ascribing deontic statuses similarly in similar cases.

I argue that the indirect commissives the courts perform are essentially indirect speech acts, in the sense that they can only be performed indirectly by way of ascribing rights in individual cases. In this sense, I agree with Timothy Endicott’s argument that the courts’ law-making acts are essentially ‘incidental’.^81^ For instance, imagine the courts make a promise as to what they will decide in the future, but that promise is irrelevant or contradictory to the ascription they make in the case before them. Then, the hearers will not consider the courts’ promise as important as when it is done by way of ascription. Or it could be better explained that the very reason of our expectation of *stare decisis* is the courts’ ability and sincerity shown by their ascription in each case. The refusal to ascribe a certain deontic status to a person in a given case is self-defeating with regard to the indirect commissive to ascribe the same type of deontic status in future similar cases. As I will explain below in subsection C, commissive acts demand the speaker’s presupposition of the speaker’s ability to perform the action they commit to. In other words, the courts’ indirect commissive acts must be performed by way of ascriptive acts in each case.

The hearers construe presumptive deontic statuses out of dispositions displayed by the courts’ past ascriptions, which in fact constitute general rules. By establishing a pattern as to when certain deontic statuses will be ascribed to individuals, the courts follow certain rules in ascribing certain deontic statuses in certain cases. In an adversarial system, the courts’ commitment to ascribing favourable statuses to one party means imposing correlative unfavourable statuses to another. Through these commitments, the courts make the hearers understand power-conferring and duty-imposing rules regarding when and how the favourable and unfavourable deontic statuses will be ascribed to individuals. Furthermore, instead of committing themselves to a future course of ascriptions in accordance with ‘judge-made’ power-conferring and duty-imposing rules, the courts can commit in accordance with a meta-rule. This meta-rule is called the rule of preference and typically contains the following three rules: ‘auctoritas superior’ (the priority of a rule created by a higher authority over one created by a lower authority); ‘auctoritas posterior’ (the priority of a rule created later over one created earlier); and ‘auctoritas specialis’ (the priority of a less general rule over a more general rule).^82^ Especially by using the first rule (auctoritas superior), the courts often choose to commit themselves to their future course of ascriptions in accordance with statutory laws.

**Figure 1:**
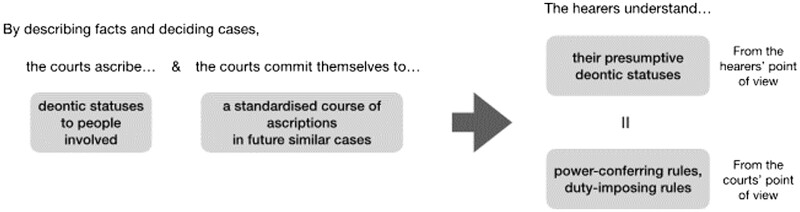
‘The structure of the courts’ speech acts’.

To sum up the discussion so far, I have argued that the courts perform indirect commissives by ascribing deontic statuses to parties in each case. When the courts perform an ascription, they indirectly commit themselves to a course of standardised future ascriptions in similar cases. By performing indirect commissives, the courts imply general rules on which they premise their own actions. These rules implied by practices include power-conferring rules, duty-imposing rules and a meta-rule of these rules, ie the rule of preference. From the viewpoint of the hearers, these general rules ground their own presumptive deontic statuses. These statuses are presumptive but fully prescriptive: the courts communicate this (commit themselves) by showing their sincerity in ascribing deontic statuses in each case. The courts can perform these commissive acts only indirectly (implicitly) by way of ascriptions in each case. See Figure 1 for the structure of the courts’ speech acts.

My account differs from the prediction theory of law in the following respects. In understanding their presumptive deontic statuses, the hearers do not simply rely on superficial regularities of the courts’ use of force. Rather, the hearers understand these statuses by the courts’ normative interventions—ascriptions. By understanding what statuses the courts ascribe to individuals, the hearers distinguish meaningful regularities (*stare decisis*) from non-meaningful regularities (eg the relationship between judgments and the judges’ breakfast). The courts demonstrate their commitment not only by showing their ability to ascribe statuses, but also by conveying their sincerity in the reasoning. The hearers, in turn, can work out the general rules by backward reasoning from the courts’ ascriptions. Presumptive deontic statuses and general rules result from the courts’ disposition towards standardised ascriptions and the hearers’ expectations of it. This is why the hearers can sometimes anticipate future ascriptions immediately after an ascription in a case.

Dicey calls the inductive process of understanding the presumptive deontic statuses *generalisation* and he calls the most fundamental of these statuses *rights*. He also appreciates that understanding such statuses involves grasping the implicit rules. As Dicey observes, ‘the law of the constitution is little else than a generalisation of the rights which the Courts secure to individuals’.^83^ The common law is the mapping of these presumptive deontic statuses, which are understood by induction from the courts’ ascription of certain deontic statuses to parties in certain cases. The protection of rights is the ascription of deontic statuses, by way of which the courts perform indirect commissives as their law-making acts. Since the courts’ law-making acts can only be performed by way of this ascription, the protection of rights is not an extra function of law which justice or law’s teleology requires, but a part of the courts’ law-making acts, without which the common law cannot exist. For the common law, the protection of rights constitutes the modality of law. I will, henceforth, focus on the courts’ indirect commissive acts to lay bare their success conditions.

### C. The Formal Requirements of the Rule of Judge-made Law

In section 2, I discussed the success conditions for Parliament’s legislative acts. Earlier in section 3, we saw that the courts’ indirect commissives as their law-making acts need to be included in law’s modality. In order to enumerate the items to add to the expanded formal requirement list, I now discuss the success conditions for the courts’ law-making acts. In doing so, I also draw a contrast between rights formalism and rulebook formalism. The success conditions for the courts’ law-making acts differ from the ones for Parliament’s legislative acts due to the different nature of their law-making acts.

For the courts to successfully commit themselves to their future actions, certain conditions must be met. The conditions I shall discuss concern two differences and one similarity between the nature of the courts’ law-making acts and Parliament’s. First, the courts’ law-making acts are *commissive*, while Parliament’s are *directive*. Second, the courts’ law-making acts are *indirect*, while Parliament’s are *direct*. Third, the success for both the courts’ and Parliament’s law-making acts require the speaker to have the relevant authority to commit the act.

I start with the first point of difference between Parliament’s law-making acts and those of the courts: *directives* versus *commissives*. Parliament’s legislative acts are commands, and therefore a type of directive, but the courts’ acts are promises, that is, a type of commissive.^84^ As the point of directives is to try to get people (not) to do something, directives generally require the *hearers*’ ability to do what is directed.^85^ In contrast, since the point of commissives is ‘to commit the speaker to doing something’,^86^ they require that the speaker assumes their *own* ability in order to be performed successfully (cf ‘I promise you the moon’).^87^ Likewise, if the courts indirectly commit themselves to something that they and the hearers clearly know the courts have no ability to do, they fail to perform this commissive act with success. The courts’ ability to ascribe deontic statuses is a matter of jurisdiction. The courts’ retaining of the full scope of jurisdiction is a foolproof way of generating the assumption of the speaker’s ability. As discussed in section 3A, equality in Dicey’s second meaning of the rule of law entails jurisdictional equality, which requires that everyone, including government officials, is under the jurisdiction of the courts.^88^ Given that the courts make general rules understood through successful commitments, being outside the jurisdiction means being outside law; where there is no jurisdiction, there is no law. Exemption from the jurisdiction creates areas of ‘lawlessness’.^89^ This lawless situation lets arbitrary power reign.^90^ The preservation of the full scope of jurisdiction indicates the speaker’s ability to conduct committed actions, which is one of the success conditions for the courts’ law-making acts.

I now turn to the second point of contrast between Parliament’s and the courts’ law-making acts: *direct* versus *indirect*. Parliament guides people’s action by explicitly bidding so. But there are several layers of indirectness in the courts’ law-making acts. The courts imply general rules on which their own actions premise *by* indirectly committing themselves to their future actions (*commissives*) *by way of* ascribing deontic statuses (by *declaratives*), which is itself first done *by way of* asserting facts (by *assertives*). Although both the courts’ and Parliament’s law-making acts eventually make general rules understood in their respective ways, the conditions for their success have different propositional content. As discussed in section 2B, being prospective is one of the propositional content conditions for parliamentary legislation. Parliament cannot command people not to have walked their dogs last week. However, this condition does not apply to the courts because they can imply future-oriented general rules by implicitly committing themselves to future actions by way of ascription in past cases. Instead, a propositional content condition for the courts to perform indirect commissives is not to contradict what they commit themselves to. A typical example of such a contradiction would be a court denying the ascription of a deontic status in the present case while trying to commit itself to future ascriptions of the same status in similar cases.

Direct and indirect law-making acts also have different success conditions with respect to the hearers’ comprehension, especially the comprehension of what the speaker *does* rather than what the speaker *says*. For Parliament’s commands, clarity of propositional content is required. But the courts not only need to make their propositional content clear; they must also establish their own precedent to ensure that the hearers comprehend the fact that the courts are engaged in committing themselves. The hearers’ comprehension of this fact is facilitated and strengthened by the repetition and habitualisation of the courts’ fulfilling their promise, ie ascribing the same deontic statuses in similar cases. The force of precedent is particularly important when the performance of indirect commissives does not rely on the use of language among the general public. Compare the following phrases:

- ‘I can φ for you’.
- ‘I can φ’.
- ‘I promise to φ’.

As seen in Searle’s example, (a) is generally understood as an indirect commissive, whose ‘indirect illocutionary act potential’^91^ is very high. This is because the speaker of (a) can rely on the general precedent other English speakers have created in using (a) for making promises indirectly. In the case of (b), however, the indirect illocutionary (speech) act potential is not so high. (b) is a simple statement of the speaker’s ability that might sometimes be understood as an indirect offer in certain contexts. But the speaker who wants to use (b) as an indirect commissive cannot completely rely on the precedent other English speakers have already established. This is because (b) can be used either to commit themselves to φ-ing in future or, in some cases, simply to show off, for example when uttered by young children. This means that the speaker who wants to use (b) as an indirect commissive needs to create his own precedent. If a person often uses this phrase without fulfilling what is supposed to be promised, people around him will start to understand that (b) is not an indirect offer but merely a case of him showing off. In this case, his precedent of breaking promises changes the function of his language, which negatively affects the hearers’ comprehension. Given the function of his language is in fact his source of power to perform indirect commissives by (b), this change means he loses the ability to perform this indirect commissive act. When this change occurs, he can no longer implicitly commit himself to a future course of action by (b) in front of his friends, who know his history of simply stating (b) to show off. In order to commit himself again successfully, he needs to use either an explicit phrase such as (c) or a phrase with higher potential such as (a). If he wants to commit himself by (b), he needs to create a precedent of using (b) as an indirect commissive from scratch, which entails the repetition of fulfilling what he promises by (b). The reliance of (b) on the speaker’s own precedent stands in contrast to (a) and the explicit version (c). In (a) and (c), breaking this promise does not usually affect the successful performance of a promise next time (even in (a), it would affect the hearers’ comprehension only if a significant portion of English speakers started to break their promises made by (a)). In (a) and (c), even when the speaker repeatedly fails to fulfil his promises, he merely becomes known as a promise-breaker.^92^ His reputation of being a promise-breaker nevertheless shows that all his *performances* of promise have been successful, as for him to break promises requires that he has made promises in the first place. This is because the use of (a) and (c) does not solely rely on the speaker’s use, so the speaker can rely on the precedent established by other English speakers’ use.

The courts’ indirect commissive acts are close to (b), since their use of language as commissives almost solely relies on the speaker’s own precedent. This is due to the several layers of indirectness in the courts’ law-making acts. The courts demonstrate their commitment *by* proving their ability to ascribe deontic statuses and their sincerity in doing so *by* actually doing so, which is again done *by way of* asserting facts and giving remedies. In order to commit themselves indirectly, the courts cannot rely on precedents created by other English speakers in their use of language.^93^ The courts need to create precedent on their own to ensure that the hearers comprehend the fact that the courts are engaged in committing themselves implicitly. This means that non-conformity to their precedent generally risks their future ability to commit themselves again at all.^94^ In other words, it puts their law-making power at risk since the precedent is the very source of power that enables the courts to perform indirect commissives. The only way for the courts to maintain their ability to perform their law-making acts (indirect commissives) is to keep fulfilling their promises, for the only way to show that their acts are promises is to fulfil them. From this perspective, it is remarkable that Dicey calls the courts’ non-conformity to precedent a ‘revolution’.^95^ By referring to non-conformity to precedent as a ‘revolution’, Dicey places this institutional commitment to the common practice accumulated as precedents on the same level as the guarantee created by written constitutions.^96^ In a country with a written constitution, the constitution cannot be legally amended except by the special procedure stipulated in its amendment clause. However, individual rights as the implicature of a written constitution can be suspended or taken away more easily than the written constitution itself, because changing the implicature does not risk the institution’s basis of power. This is because, in this case, the ultimate source of power lies in the written constitution, unless they change the interpretation of the very clause authorising themselves or of the amendment clause.^97^ For a judge-made constitution, however, the courts’ power to legislate lies in their respect for and leveraging of precedent, which is the requirement for ensuring the hearer’s comprehension of their indirect commissives. This is why an unwritten constitution can be as secure as, or even more secure than, written constitutions. In praise of the British constitution, Dicey notes that where the practice of giving remedies against violations of individual freedom is firmly established (eg by habeas corpus in the UK), *the constitution as the implicature* of this institutional practice can also be so firm that only ‘a thorough revolution in the institution and manners of the nation’ could destroy it.^98^ The indirectness of their law-making acts requires the courts to follow precedent in order to maintain their ability to commit themselves again, which is the foundation of their power of law making. Breaking the precedent up altogether amounts to a revolution, which overturns the source of the courts’ political power to make law.

The courts need to find like cases for opportunities to show that they are fulfilling what they have promised. This approach makes the courts’ laws general and stable to a greater extent than Parliament’s law-making acts. The risk of losing the source of power to perform indirect commissives through non-conformity to precedent also means that there is a relative difficulty in distinguishing previous decisions. If the courts were to distinguish in every case, this would amount to the non-existence of precedent, which would mean that the courts could not perform indirect commissives successfully. Given that the courts can only fulfil their promises in like cases, the hearers will never be able to recognise that the courts are actually promising indirectly if the courts were to always make exceptions. If the courts were to make exceptions in every case, they were like a person who never keeps a promise when the presenting situation is somewhat different from what was envisaged at the time of making the promise. In the case of the courts, this practice would cost them the ability to commit themselves in the first place, since the hearers know the courts are engaged in promising only by the fact that the courts fulfil their promises. In other words, the courts can distinguish the case at hand from past cases, but their ability to do so weighs against their need to abide by precedent insofar as consistency *vis-à-vis* precedent is required to maintain law-making powers. The courts need to *find* ‘like cases’ to prove that they are treating like cases alike. Synchronically, finding like cases broadens the classes of cases and people to which a rule or a principle is applied (Dicey’s equality as generality). Diachronically, it leads to the stability of judge-made law through time.^99^ In Parliament’s case, they can more flexibly particularise or change their commands because their law-making acts are explicit. Due to the combination of indirectness and commissiveness, the successful performances of the courts’ law-making acts are likely to entail a greater level of generality and stability of the rules the courts create. This restraint also means that the courts must follow the rules they make because that is the only way for them to continue making laws. These facts reflect the by-productive virtue resulting from the courts’ successfully performing law-making acts.

Finally, I shall consider the requirement of the speaker’s authority. Parliament guides people’s actions by invoking its authority. The courts also need authority in order to perform their indirect commissives with success. This is required for the following two reasons. First, as discussed in section 3B, the courts’ law-making acts, as indirect commissives, can only be performed by way of ascribing deontic statuses to people involved in each case. Therefore, the success conditions for their indirect commissives are interlocked with the success conditions for their ascriptions. The declarative’s mode of achieving its characteristic aim, ‘changing the world by saying so’, requires that ‘the speaker invokes his power or authority to perform’ them. Thus, declaratives require that ‘the speaker has that power or authority’ as a general preparatory condition.^100^ The authority to ascribe deontic statuses, especially rights and duties, lies in the ordinary courts. Second, as we have seen, due to their acts being indirect commissives, the speaker needs to have established their precedent to give the commissive function to their language.^101^ The ordinary courts have established this precedent throughout the course of their history, but it is not often the case for other, newer tribunals. The ordinary courts are by far the best speaker to perform indirect commissives. One of the most criticised aspects of Dicey’s concept of the rule of law has been its unusual emphasis on ‘the ordinary tribunals’.^102^ But this ordinary tribunal requirement can be understood as a success condition of the courts’ law-making acts.

In sum, the success conditions for the courts’ law-making acts include:

- that the ordinary tribunal meets the speaker’s authority requirements;
- the preservation of the full scope of jurisdiction as a requirement of the speaker’s ability to conduct the committed action; and
- the force of precedent as a requirement of the hearers’ comprehension.

When these formal requirements are satisfied, it is also likely that laws have the following virtues: they are general and stable, and are followed by the courts. These virtues are necessitated by their law-making acts being indirect commissives. Furthermore, in general, when the courts’ law-making acts are successfully performed, they are likely to entail these virtues to a greater degree than Parliament’s law-making acts do, due to their law-making acts’ different natures. See Table 1 for a comparison between rights formalism and rulebook formalism.

**Table 1:** The formal requirements of the rule of judge-made law and legislated law

	The formal requirements of the rule of law	
	The rule of judge-made law (Rights formalism)	The rule of legislated law (Rulebook formalism)
Law-making act	Indirect commissive by way of ascribing individual rights	Command
The speaker’s authority	The authority of ordinary tribunals	Promulgation
The speaker’s ability	Retaining the full scope of jurisdiction	N/R
The hearers’ ability	N/R	Prospectivity, consistency
The hearers’ comprehension	The force of precedent	Clarity, promulgation

## 4. Conclusion

The list of formal requirements defined by Fuller’s desiderata is best understood against the backdrop of the list of the success conditions for command-type law-making acts — the formal requirements of the rule of legislated law. However, commands are merely one way of making laws, and there are different ways of making general rules understood. Fuller’s list is, therefore, incomplete. In order to factor the common law into the rule of law, it is important to introduce a different type of law-making act, the implicit type. The courts make general rules understood through their indirect commissive acts. The courts’ law-making acts postulate different conditions and features of law as law’s modality (rights formalism). The combination of the indirect and commissive nature of the courts’ law-making acts creates a peculiar environment for the rule of law, where, for the courts’ law-making acts to satisfy the relevant success conditions, the courts must follow precedent. The courts’ law-making acts may make rules more general and stable than Parliament’s law-making acts do. This, in turn, means that the courts are bound by the general rules they themselves created. The protection of rights is the courts’ ascription of deontic statuses to particular persons, without which the courts cannot perform their law-making acts. The higher level of generality in law, the protection of rights and the courts’ being bound by precedent are not the law’s own end or the requirements of justice. They are the virtue arising by-productively from the different mode of making the common law compared to legislation. We can agree with the formality of the rule of law by acknowledging that there are necessary features of law as its modality that are postulated by law-making acts. However, there are several ways to perform law-making acts in our actual world—in the case of Parliament, by commands, and in the case of the courts in the UK, by commissives. They have different success conditions and therefore necessitate different items in the list — the formal requirements of the rule of judge-made law. These requirements are not the substance of the rule of law or justice or any teleological arguments. The emphasis on the form of the rule of law in Raz-Fuller’s framework requires this variant list, as long as we acknowledge the common law as the law and the peculiarities of its law-making acts.

## Footnotes

1 Lon Fuller, *The Morality of Law* (Yale UP 1977).

2 Joseph Raz, ‘The Rule of Law and Its Virtue’ in *The Authority of Law: Essays on Law and Morality* (OUP 1979) ch 11.

3 Joseph Raz, ‘The Law’s Own Virtue’ (2019) 39 OJLS 1.

4 Fuller (n 1) 41 et seq.

5 Raz, ‘The Rule of Law and Its Virtue’ (n 2) 214.

6 ibid.

7 ibid; Paul Craig, ‘Formal and Substantive Conceptions of the Rule of Law: An Analytical Framework’ [1997] PL 467; Jeremy Waldron, ‘The Concept and the Rule of Law’ (2008) New York University School of Law: Public Law & Legal Theory Research Paper Series Working Paper No 08-50, 6; NW Barber, ‘Must Legalistic Conceptions of the Rule of Law Have a Social Dimension?’ (2004) Ratio Juris 474; Neil MacCormick, ‘Natural Law and the Separation of Law and Morals’ in Robert P George (ed), *Natural Law Theory: Contemporary Essays* (OUP 1992) ch 5, 121; Andrei Marmor, ‘The Rule of Law and Its Limits’ (2004) 23 Law and Philosophy 1. See also Joseph Raz, ‘Formalism and the Rule of Law’ in George (ibid) ch 11, which is to be read in relation to Ernest J Weinrib, ‘Why Legal Formalism’ in the same book ch 12.

8 Raz, ‘The Law’s Own Virtue’ (n 3) 226.

9 John Gardner, ‘The Supposed Formality of the Rule of Law’ in *Law as a Leap of Faith* (OUP 2012) ch 8.

10 Leslie Green, ‘The Concept of Law Revisited’ (1996) 94 Mich L Rev 1687, 1710.

11 As to the view that law is a social artefact, see also Timothy Endicott, ‘The Generality of Law’ in Luís Duarte d’Almeida, James Edwards and Andrea Dolcetti (eds), *Reading HLA Hart’s ‘The Concept of Law’* (Hart Publishing 2014) ch 2; Brian Leiter, The Demarcation Problem in Jurisprudence: A New Case for Scepticism (2011) 31 OJLS 663.

12 Green (n 10) 1710.

13 ibid 1711.

14 As the background behind the distinction between a modal and functional kind, there were debates on biological functions and artefact functions in philosophy in the latter half of the 20th century. One of the classical works is Carl Hempel, ‘The Logic of Functional Analysis’ in *Aspects of Scientific Explanation and Other Essays in the Philosophy of Science* (New York: Free Press 1965) ch 11. For a discussion of functional analysis in etiology and its analogs in law, see Michael S Moore, ‘Law as a Functional Kind’ in George (n 7) ch 8. However, given the development in recent literature on this subject, emphasising the fundamental difference between artefact function and biological function is misleading. The ascription of biological function can rely on the notion of Darwinian selection, which in fact plays a similar role to the pre-Darwinian perspective, ie the Creator’s intention. Regarding the account of biological functions in terms of biological capacities, see Robert Cummins, ‘Functional Analysis’ (1975) 72 Journal of Philosophy 741. See also Philip Kitcher, ‘Function and Design’ (1993) 18 Midwest Studies in Philosophy 379; Bence Nanay, ‘A Modal theory of Function’ (2010) 107 The Journal of Philosophy 412 argues that function analysis in etiology, the same as the function analysis of artifact, is modal analysis. See also Ruth Garrett Millikan, *Language, Thought, and Other Biological Categories: New Foundations for Realism* (The MIT Press 1984).

15 JL Austin called actions performed by uttering words speech acts or illocutionary acts: JL Austin, *How to Do Things with Words* (OUP 1962).

16 cf Jeremy Bentham, ‘Of Laws in General’ (HLA Hart ed, University of London Press 1970) ch 5. For a view of permissions as the subtraction of norms created by command, see Eugenio Bulygin and Carlos E Alchourrón, ‘The Expressive Conception of Norms’ in Eugenio Bulygin and others, *Essays in Legal Philosophy* (Carlos Bernal and others eds, OUP 2015) ch 9.

17 John Searle, ‘A Taxonomy of Illocutionary Acts’ in *Expression and Meaning: Studies in the Theory of Speech Acts* (CUP 1999) ch 1, 13. As to the characteristic aim of a speech act, see also John R Searle and Daniel Vanderveken, *Foundations of Illocutionary Logic* (CUP 1985) 13.

18 Searle and Vanderveken call it the ‘modes of achievement of their illocutionary point’: Searle and Vanderveken (n 17) 16.

19 ibid 15.

20 This contrasts with the case of persuading, in which the success of the directive might depend on the content of the utterance in addition to the social position of the speaker. John R Searle, ‘The Structure of Illocutionary Acts’ in *Speech Acts: An Essay in the Philosophy of Language* (CUP 1969) ch 3, 54. Daniel Vanderveken explains this by the idea of ‘self-defeating speech acts’: Daniel Vanderveken, ‘Illocutionary Logic and Self-Defeating Speech Acts’ in JR Searle and others eds, *Speech Act Theory and Pragmatics* (D Reidel 1980) 247. cf Austin’s discussion of purported speech acts that are void, often because of the lack of a putative procedure or authority (eg trying to marry a couple without any authority to do so). Speech acts can misfire if they are performed, in Austin’s term, *infelicitously*. Austin (n 15) Lecture II.

21 Fuller (n 1) 49.

22 Searle and Vanderveken (n 17) 15. This fact also determines the unique ‘degree of strength’ in achieving its characteristic aim.

23 ibid 65, 70.

24 Craig (n 7) 467.

25 Fuller (n 1) 63.

26 My use of the terms, ‘rulebook’ versus ‘rights’ comes from Ronald Dworkin’s use in his *A Matter of Principle* (Harvard UP 1985) 11 et seq. However, as I explain later in this article, I construe ‘rights’ conceptions in Raz’s framework rather than substantive justice (ibid 12).

27 AV Dicey, *Lectures Introductory to the Study of the Law of the Constitution* (JWF Allison ed, OUP 2013) 97, 225.

28 ibid.

29 ibid 100, 228.

30 ibid.

31 ibid.

32 ibid 115, 229.

33 Craig (n 7) 471.

34 ibid.

35 ibid 473.

36 ibid. By arguing so, Craig goes in the opposite direction of Dworkin, who claims that the rule of law as substantive justice requires protecting citizen’s moral rights against the state: Dworkin (n 27) 11, 12.

37 Dicey, *Lectures Introductory* (n 28) 435 (emphasis added). The legislation Dicey criticises includes the National Insurance Acts (1911) and (1913), which marked, in Craig’s term, ‘the birth of the welfare state and the development of the tribunal system’: Paul Craig, *Administrative Law* (8th edn, Thomson Reuters 2016) 2-017. As to the criticism, see also HW Arthurs, ‘Rethinking Administrative Law: A Slightly Dicey Business’ (1979) 17 Osgoode Hall LJ 1; William A Robson, ‘Dicey on the Constitution’ (1939) 38 Mich L Rev 205, 207.

38 This term was coined by Timothy Endicott, *Vagueness in Law* (OUP 2000) ch 9, 194.

39 JWF Allison, *The English Historical Constitution: Continuity, Change and European Effects* (CUP 2007) 159). This also concerns Dicey’s notorious comparison between the British system and the French system. Due to his criticism of *droit administratif*, as Lord Bingham observes, ‘Dicey’s reputation has suffered gravely’: Lord Bingham of Cornhill, ‘Dicey Revisited: The Rule of Law’ [2002] PL 39, 51.

40 AV Dicey, *Lectures on the Relation between Law and Public Opinion in England during the Nineteenth Century* (2nd edn, Macmillan 1930) 488.

41 In Trevor Allan’s words, it is equality of ‘the application of a substantive principle’: TRS Allan, *Constitutional Justice: A Liberal Theory of the Rule of Law* (OUP 2001) 18.

42 cf Endicott, ‘The Generality of Law’ (n 11) 27–9.

43 Fuller (n 1) 47.

44 Gardner (n 9) 210.

45 Fuller (n 1) 81.

46 ibid.

47 Raz, ‘The Law’s Own Virtue’ (n 3) 4.

48 ibid.

49 ibid 5.

50 ibid 9.

51 ibid 7. He also acknowledges that his new list still ‘does not guarantee that the law is good or just’ (ibid).

52 As Endicott points out, even when statutory law is indeterminate, the courts often need to resolve disputes ‘under the law of the jurisdiction’. Timothy Endicott, ‘Interpretation and Indeterminacy’ (2014) 10 Jerusalem Rev Leg Stud 46, 55.

53 HLA Hart, *The Concept of Law* (3rd edn, OUP 2012) 141.

54 Thomas Erskine Holland, *The Elements of Jurisprudence* (12th edn, Clarendon Press 1916) 65.

55 William Blackstone, *Commentaries of the Laws of England*, vol 1 (JB Lippincott 1893) Introduction, s III, 69.

56 Holland (n 55) 65–6.

57 John Austin, *Lectures on Jurisprudence or the Philosophy of Positive Law*, vol 2 (John Murray 1911) 634. See also John Finnis, *Philosophy of Law: Collected Essays*, vol IV (OUP 2011) ch 20, 400.

58 Holland (n 55) 66.

59 Dicey, *Lectures on the Relation* (n 41) 484.

60 Austin (n 58) 634.

61 AV Dicey, ‘Can English Law be Taught at the Universities?’ in Dicey, *Lectures Introductory* (n 28) Appendix I.

62 Dicey, *Lectures Introductory* (n 28) 117 (emphasis added).

63 The current terminology distinguishes induction from abduction, a type of induction in a broad sense. In the current terminology, Dicey’s ‘induction’ is called abduction.

64 Neil MacCormick, ‘Legal Deduction, Legal Predicates and Expert Systems’ (1992) 5 International Journal for the Semiotics of Law 181, 182.

65 AV Dicey, ‘Introduction to the Study of English Law’ (1902) 8 Working Men’s College Journal 256, 257.

66 ibid. See also Timothy Endicott, ‘How Judges Make Law’ in Liz Fisher, Jeff King and Alison Young (eds), *The Foundations and Future of Public Law* (OUP 2020) ch 7, 134–5. Furthermore, as the courts have the authority to give the interpretation of statutory laws, Dicey holds that law in general needs to be identified by inductions. He defines a law as ‘a rule of human action or conduct *recognised as binding* by the Courts’: AV Dicey, ‘Introduction to the Study of English Law’ (1901) 7 Working Men’s College Journal 168 (emphasis original).

67 Hans Kelsen, *General Theory of Law and State* (Harvard UP 1949) 132. By regarding the court decisions as an act of creating individual norms, Kelsen also shows that there is merely a relative difference between what we call the creation and application of law. The act of application is the act of creation. It is a voluntary act, not solely a cognitive act (ibid 132). As to Kelsen’s creation of individual norms, see also Bulygin (n 16) ch 4, 79; Endicott (n 67), especially s III. Kelsen himself does not deny the creation of general norms from individual norms, but he criticises John Chipman Grey’s idea that ‘all the law is judge-made law’. For Kelsen, securing the flow of norm-creation from general norms to individual norms is essential for the continental constitutional system: Kelsen (ibid) 150 et seq. In this respect, Dicey’s idea is closer to Grey’s than Kelsen’s.

68 Dicey, *Lectures Introductory* (n 28) 118.

69 ibid 117.

70 HLA Hart, ‘The Ascription of Responsibility and Rights’ (1948) 49 Proceedings of the Aristotelian Society 171, 171. The ascription of rights is, in Kelsen’s term, the imputation of norms. However, as Kelsen’s term, ‘imputation’, carries a sound of pre-existing general norms, I prefer to use Hart’s term, ‘ascription’. For criticism of this view, see George Pitcher, ‘Hart on Action and Responsibility’ (1960) 69 The Philosophical Review 226; PT Geach, ‘Ascriptivism’ (1960) 69 The Philosophical Review 221; PT Geach, ‘Assertion’ (1965) 74 The Philosophical Review 449. While Pitcher criticises Hart, Geach’s criticism is not directed specifically towards Hart but towards the old expressivists’ (emotivists’) contention that moral judgments cannot be true or false. As to Geach’s criticism against old expressivism, see James Dreier, ‘Expressivist Embeddings and Minimalist Truth’ (1996) 83 Philosophical Studies 29. Facing their criticism, Hart decided not to include the above-mentioned article in his book *Punishment and Responsibility* and says that ‘My reason for excluding it is simply that its main contentions no longer seem to me defensible, and that the main criticisms of it made in recent years are justified’: HLA Hart, *Punishment and Responsibility: Essays in the Philosophy of Law* (1st edn, OUP 1968) Preface. Searle discusses the problem when he criticises ‘speech act fallacy’. For Searle, the analysis in early speech act theory, including JL Austin’s and early expressivists’, is inadequate in the sense that they tend to present their analysis as if it were the analysis of the meaning of the words used: Searle, *Speech Acts* (n 20) 136–7. Such an analysis is inadequate for speech acts due to the essentially different nature of the analysis of words and the analysis of remarks: while the analysis of words is to be applied to all occurrences of the words, for a speaker, it is inappropriate to assert something without anything ‘non-standard or abnormal’ (eg the oddity of asserting ‘He is breathing’ without any special circumstances to assert so). As Searle says, ‘The point is not, “No modification without aberration”, but “No remark without remarkableness”’: ibid 143–4. Arising from old expressivism, the recent expressivists emphasise their positive argument in favour of the distinctive *functional roles* of normative expressions. Regarding the overview and criticism of the development in recent expressivism, see James Dreier, ‘Meta-ethics and the Problem of Creeping Minimalism’ (2004) 18 Philosophical Perspectives 23. As for the defence of expressivism regarding creeping minimalism, see also David Macarthur and Huw Price, ‘Pragmatism, Quasi-realism, and the Global Challenge’ in Cheryl Misak (ed), *New Pragmatists* (OUP 2007) ch 5. Given the recent debates on expressivism after Hart, his earlier article is very defensible. Pitcher’s and Geach’s criticisms take Hart’s analysis as the analysis of *words* and the *concept* of responsibility. However, as Hart himself made clear at the beginning of his article, his point was to emphasise the distinctive ‘function’ of sentences when they are used ‘in the practice and procedure of the law’, and such an inquiry differs from ‘the theoretical discussions of legal concepts by jurists who are apt to be influenced by philosophical theories’: Hart (ibid) 172. Despite early criticisms, I will take Hart’s early analysis as the functional analysis of normative expression, as distinct from the analysis of words and concepts.

71 Searle (n 17) 16.

72 Searle and Vanderveken (n 17) 37.

73 Searle (n 17) 12.

74 This means, the act of ascription is an indirect speech act. See Searle, ‘Indirect speech acts’ in *Expression and Meaning* (n 17) ch 2, 30.

75 Dicey follows Holland in taking rights, rather than duties, as the starting point for analysing a legal system. cf Holland (n 55) 88. Here, rights include liberties (Dicey, ‘Introduction to the Study of English Law’ (n 66) 168). It is unimportant for them whether to start with rights or duties since, in Dicey’s terms, ‘with every legal right there existed a corresponding duty’ (ibid). Hohfeld argues that Holland’s usage of rights (and therefore Dicey’s usage, too) is all-inclusive and ‘the notions of right, privilege, and power seem to be blended’: Wesley Newcomb Hohfeld, *Fundamental Legal Conceptions as Applied in Judicial Reasoning* (David Campbell and Philip Thomas eds, Dartmouth Publishing 2001) 14, 15.

76 Dicey, ‘Introduction to the Study of English Law’ (n 66) 168. He adopted this definition from Holland (n 55) 82, 83.

77 Searle, ‘Indirect speech acts’ (n 75) 54. Searle discusses *indirect directives* (eg ‘Can you pass me the salt?’) and *indirect commissives* (eg ‘Can I do this for you?’) as two major indirect speech acts.

78 Searle (n 75) 30.

79 ibid.

80 ibid 55.

81 Endicott (n 67).

82 Bulygin and Alchourrón (n 16) 158, 159.

83 Dicey, *Lectures Introductory* (n 28) 118.

84 Searle (n 17) 13, 14. As I explained in s 2B, the courts’ indirect commissives also imply duty-imposing rules. As Searle acknowledges, commissives and directives are similar as both assume obligation. However, directives and commissives communicate this obligation differently. While directives imply obligation by trying to get the hearers to do what is prescribed, commissives imply obligation by committing the speaker to an ascription. The courts make the hearers understand obligations, but they do so by implying general rules on which their own actions are premised. This difference leads to the different success conditions between the courts’ and Parliament’s law-making acts.

85 Fuller (n 1) 71. He uses the example of a dictator’s absurd order that the subjects should keep themselves ‘ready to jump in any direction’.

86 Searle (n 17) 14.

87 Searle and Vanderveken (n 17) 17.

88 Dicey, *Lectures Introductory* (n 28) 100, 108.

89 ibid 435. As mentioned in s 3A, Dicey calls the legislations ousting the courts’ jurisdiction ‘lawless methods’.

90 ibid 98, 99.

91 Searle, ‘Indirect speech acts’ (n 75) 49.

92 People might start to doubt the sincerity of the speaker, but sincerity itself is not an independent success condition of promises. See ibid. Searle isolates the sincerity condition from the success conditions and categorises it as a condition for non-defectiveness.

93 The courts, of course, need to rely on English grammar and other English speakers’ usage to convey the propositional content of their commitment. But what I am discussing here is what the courts can *do* with this propositional content and how the hearers can understand what the courts *are doing*. The indirectness lies not in the propositional contents of what the courts are saying, but in what the courts *are doing*.

94 When the courts change their actions in response to statutory law revisions, this problem does not arise, as the courts are conforming to a meta-rule, the rule of preference, which requires the courts to follow parliamentary statutes. Problematic changes (such as non-conformity to judicial precedent) instead take place when the courts change their own interpretation of statutory law or judge-made law independent of Parliament. As to whether this non-conformity creates a new ‘heroic case’ or one of the countless cases which law students cease to read, see Endicott (n 67) s VII.

95 Dicey, *Lectures Introductory* (n 28) 118.

96 ibid.

97 For this reason, Alf Ross argues that the amendment of an amendment clause in a written constitution is logically unexplainable, unless, like Kelsen, we assume a higher basic norm as the ultimate source of legal power: Alf Ross, ‘On Self-Reference and a Puzzle in Constitutional Law’ (1969) 78 Mind 1. HLA Hart criticises Ross’s position in *Essays in Jurisprudence and Philosophy* (OUP 1983) ch 7.

98 Dicey, *Lectures Introductory* (n 28) 118.

99 In the writing of his later years, Dicey says, in a slightly cynical tone, ‘judge-made law occasionally represents the opinion of the day before yesterday’ (Dicey, *Lectures on the Relation* (n 41) 369). As to the stability, see also Marmor, ‘The Rule of Law and Its Limits’ (n 7) 34.

100 Searle and Vanderveken (n 17) 57.

101 Marmor argues that the role of the convention ie the rule of recognition, is constitutive. Andrei Marmor, ‘The Conventional Foundations of Law’ in Stefano Bertea and George Pavlakos eds, *New Essays on the Normativity of Law* (Hart Publishing 2011) ch 5. cf Julie Dickson, ‘Is the Rule of Recognition Really a Conventional Rule?’ (2007) 27 OJLS 373.

102 Dicey, *Lectures Introductory* (n 28) 100.

## Notes

I am grateful to Timothy Endicott, Trevor Allan, Alison Young, Jonah Goldman, Mariama Sheriff, Lars Vinx, and Matthew Kramer for extraordinarily helpful comments at various stages of writing this article.

